# Dose-response relationship between the triglyceride-glucose index and metabolic syndrome risk in patients with type 2 diabetes: a cross-sectional analysis of 1,560 cases

**DOI:** 10.3389/fendo.2025.1615380

**Published:** 2025-09-12

**Authors:** Feng Chen, Zixue Gu, Rong Peng

**Affiliations:** ^1^ School of Basic Medical Sciences & School of Nursing, Chengdu University, Chengdu, Sichuan, China; ^2^ Clinical Medical College & Affiliated Hospital of Chengdu University, Chengdu University, Chengdu, Sichuan, China

**Keywords:** triglyceride glucose index, type 2 diabetes mellitus, metabolic syndrome, overweight or obesity, dose-response relationship

## Abstract

**Background:**

The triglyceride-glucose (TyG) index, as a novel biomarker for assessing insulin resistance, may possess predictive value for metabolic syndrome (MS) in patients with type 2 diabetes mellitus (T2DM). However, its dose-response relationship requires further investigation. Therefore, this cross-sectional study aimed to examine the association between the TyG index and MS, along with their dose-response relationship, in a T2DM population.

**Methods:**

This cross-sectional study enrolled patients with T2DM from a tertiary hospital in Chengdu between January 2018 and December 2023. Participants were stratified into quartiles (Q1-Q4) based on TyG index levels. We employed multivariable logistic regression to analyze associations between TyG index and MS and its components. Predictive performance was evaluated using receiver operating characteristic (ROC) curve analysis, while restricted cubic spline analysis was utilized to examine the dose-response relationship between TyG index and MS.

**Results:**

This study included a total of 1,560 patients with T2DM. With increasing TyG index levels, patients were significantly younger, had lower HDL-C levels, higher rates of current alcohol consumption, and elevated BMI, TG, TC, LDL-C and FPG values (all P<0.05). The prevalence of MS, overweight/obesity and dyslipidemia also progressively increased with higher TyG quartiles (all P<0.05). Pearson correlation analysis showed the TyG index was positively correlated with BMI, TG, TC, LDL-C and FPG (all P<0.001), while negatively correlated with HDL-C (P<0.001). After adjusting for confounding factors, multivariate logistic regression analysis revealed that compared with the Q1 group, the Q4 group had a significantly higher risk of MS (OR=26.994), overweight/obesity and dyslipidemia (all P<0.001). The area under the curve (AUC) for TyG index in predicting MS was 0.793,with a sensitivity of 0.864 and a specificity of 0.611. Furthermore, a nonlinear dose-response relationship was observed between TyG index and MS, with MS risk increasing progressively when TyG index exceeded 9.31.

**Conclusion:**

The TyG index serves as an independent predictor of MS risk in patients with T2DM, demonstrating a significant dose-response relationship with MS.

## 1 Introduction

Metabolic syndrome (MS) is a cluster of metabolic disorders characterized primarily by insulin resistance (IR), typically manifesting as central obesity, impaired glucose tolerance, hypertension, and dyslipidemia - a confluence of multiple cardiovascular risk factors (1–3). Epidemiological data indicate that MS affects 20%-25% of the adult population (4). MS not only accelerates disease progression in individuals with type 2 diabetes mellitus (T2DM), significantly increasing their risk of cardiovascular diseases and diabetes-related complications (5–8), but also imposes a substantial socioeconomic burden (9). Consequently, early identification of high-risk MS populations holds crucial clinical significance for the comprehensive management of T2DM patients.

Accurate assessment of IR is crucial for early intervention in metabolic syndrome. Although the hyperinsulinemic-euglycemic clamp remains the gold standard for diagnosing IR, its clinical utility is limited by procedural complexity and high costs (10, 11). The recently proposed triglyceride-glucose (TyG) index has emerged as a promising IR biomarker, demonstrating excellent clinical applicability due to its strong correlation with gold-standard methods (12). Multiple studies have shown that the TyG index outperforms the conventional HOMA-IR index in predicting MS risk (13), while exhibiting a significant dose-response relationship with MS components (14).

However, current large-scale studies investigating the association between the TyG index and MS in T2DM patients remain insufficient, particularly lacking in-depth analyses of Chinese populations. To address this gap, this retrospective study analyzed clinical data from 1,560 T2DM patients to: (1) elucidate the correlation between the TyG index and MS and its components, and (2) explore the dose-response relationship between the TyG index and MS risk. The findings will provide evidence-based support for early screening and stratified management of MS in T2DM patients.

## 2 Materials and methods

### 2.1 Study participants

This study is a cross-sectional study, retrospectively enrolling patients with T2DM who visited a tertiary hospital in Chengdu from January 2018 to December 2023. Inclusion criteria: (1) Age > 18 years; (2) Meeting the diagnostic criteria for T2DM established by the World Health Organization in 1999 (15); (3) Having complete clinical data. Exclusion criteria: (1) Women who are breastfeeding or pregnant; (2) Patients with malignant tumors. This study was approved by the Ethics Committee of the Affiliated Hospital of Chengdu University (Approval No. PJ2024-081-01).

### 2.2 Methods

We systematically collected baseline clinical characteristics of all participants, including gender, age, smoking status, alcohol consumption history, dyslipidemia history, and hypertension history. All physical examinations and laboratory tests were performed by certified medical professionals. We measured resting diastolic blood pressure (DBP) and systolic blood pressure (SBP), recorded body weight and height to calculate body mass index (BMI = weight [kg]/height [m]²). Following an overnight fast of ≥8 hours, 5 mL of fasting venous blood was collected from the antecubital vein on the second morning of hospitalization. Biochemical analyses including fasting plasma glucose (FPG), triglycerides (TG), total cholesterol (TC), high-density lipoprotein cholesterol (HDL-C), and low-density lipoprotein cholesterol (LDL-C) were performed using a Beckman Coulter AU5800 automated biochemical analyzer in our hospital’s central laboratory. The triglyceride-glucose (TyG) index was calculated using the formula: TyG index = Ln (fasting triglycerides [mg/dL] × fasting glucose [mg/dL])/2 (16).

### 2.3 Diagnostic criteria

The diagnostic criteria for MS were based on the standards set by the Chinese Diabetes Society: the presence of any three or all of the following criteria indicates MS (1): (1) Hypertension: SBP/DBP ≥ 140/90 mmHg, or a confirmed diagnosis of hypertension with treatment; (2) Dyslipidemia: fasting TG ≥ 1.7 mmol/L, or fasting HDL-C < 0.9 mmol/L (male) or < 1.0 mmol/L (female); (3) Hyperglycemia: FPG ≥ 6.1 mmol/L, or postprandial two-hour glucose ≥ 7.8 mmol/L, or a confirmed diagnosis of diabetes with treatment; (4) Overweight or obesity: BMI ≥ 25 (kg/m²).

### 2.4 Statistical analysis

Normally distributed data were expressed as mean ± standard deviation (SD), and intergroup comparisons were performed using one-way analysis of variance (ANOVA). Non-normally distributed data were presented as median (interquartile range) [M (P25, P75)], with group differences assessed by the Kruskal-Wallis H test, followed by pairwise Mann-Whitney U tests for *post hoc* analysis. Categorical variables were described as frequency (percentage) [n (%)], and intergroup differences were evaluated using the chi-square test (χ²).Pearson correlation analysis was employed to quantify the linear association between the TyG index and metabolic parameters (e.g., BMI, blood lipids, glucose levels). To account for potential confounding factors, partial correlation analysis was further conducted, adjusting for covariates such as sex, age, smoking status, and alcohol consumption, with adjusted correlation coefficients and their 95% confidence intervals (CIs) reported. Multivariable logistic regression models were constructed to assess the relationship between TyG index quartiles (Q1-Q4, as the independent variable) and MS and its components (overweight/obesity and dyslipidemia, as dependent variables). The models were adjusted for confounders in three sequential steps, with results expressed as odds ratios (ORs) and 95% CIs. Trend P-values were calculated to evaluate dose-response relationships. The predictive performance of the TyG index for MS was evaluated using receiver operating characteristic (ROC) curve analysis, and the area under the curve (AUC) was computed. Restricted cubic splines (RCS) models were used to model the relationship between the TyG index and metabolic syndrome. The goodness-of-fit of a linear model was compared with that of a non-linear model using a likelihood-ratio test, with inflection points and their 95% CIs estimated. All statistical analyses were performed using SPSS 26.0 and R 4.4.2. A two-sided P-value < 0.05 was considered statistically significant.

## 3 Results

### 3.1 Baseline characteristics of the study participants

This study enrolled 1,560 patients with type 2 diabetes mellitus (T2DM), including 1,028 males (65.9%) and 532 females (34.1%), with a mean age of 59.42 ± 11.76 years. Participants were stratified into quartiles (Q1-Q4) based on TyG index levels: Q1 (TyG ≤ 8.83), Q2 (8.83<TyG ≤ 9.31), Q3 (9.31<TyG ≤ 9.87), and Q4 (TyG>9.87).Significant metabolic differences were observed across TyG quartiles. With increasing TyG levels, participants demonstrated progressively worsening metabolic profiles: younger age, elevated BMI, TG, TC, LDL-C, and FPG (all P<0.001). Conversely, HDL-C levels significantly decreased with higher TyG quartiles, while current alcohol consumption rates increased markedly. Notably, the prevalence rates of MS, overweight/obesity, and dyslipidemia showed significant stepwise increases across TyG quartiles (all P<0.001). In contrast, no significant differences were found in gender distribution, smoking status, blood pressure levels (DBP and SBP), or hypertension prevalence among TyG groups (all P>0.05) **Table 1**.

**Table 1:** Baseline characteristics of the study participants.

Baseline Characteristics	Q1 (n=390)	Q2 (n=390)	Q3 (n=390)	Q4 (n=390)	*χ*²/*F*/*H*	*P* ^##^
Age (years)*	63.63 ± 11.11	61.21 ± 10.50	57.96 ± 11.53	54.88 ± 11.99	44.507	<0.001^a^
Male**	257 (65.90)	252 (64.62)	253 (64.87)	266 (68.21)	1.392	0.707
Current Smoking**	79 (20.26)	85 (21.79)	83 (21.28)	105 (26.92)	5.929	0.115
Current Drinking**	78 (20.00)	76 (19.49)	90 (23.08)	105 (26.92)	7.895	0.048
DBP (mmHg)*	71.46 ± 9.98	72.33 ± 9.65	72.53 ± 10.22	73.22 ± 11.25	1.774	0.15
SBP (mmHg)*	126.83 ± 12.99	127.02 ± 12.90	126.73 ± 12.26	126.98 ± 13.57	0.041	0.989
BMI (kg/m²)^#^	23.5 (21.6,25.6)	24.6 (22.3,26.6)	25.4 (23.4,27.5)	26.5 (23.8,28.9)	145.637	<0.001^a^
TG (mmol/L)^#^	0.94 (0.76,1.11)	1.41 (1.19,1.66)	1.98 (1.68,2.41)	3.67 (2.62,5.46)	1122.488	<0.001^a^
TC (mmol/L)^#^	4.32 (3.58,4.96)	4.60 (3.84,5.27)	4.9 (4.23,5.61)	5.24 (4.48,6.03)	145.868	<0.001^a^
HDL-C (mmol/L)^#^	1.39 (1.16,1.67)	1.29 (1.08,1.54)	1.21 (1.03,1.45)	1.07 (0.87,1.23)	221.978	<0.001^b^
LDL-C (mmol/L)*	2.36 ± 0.82	2.64 ± 0.81	2.80 ± 0.80	2.81 ± 0.90	24.348	<0.001^c^
FPG (mmol/L)^#^	6.30 (5.35,7.26)	7.67 (6.61,8.86)	8.71 (7.45,10.44)	11.22 (8.72,15.11)	610.387	<0.001^a^
MS**	23 (5.89)	72 (18.26)	175 (44.87)	239 (61.28)	334.638	<0.001^d^
Overweight or Obesity**	122 (31.28)	182 (46.47)	213 (54.62)	250 (64.10)	90.33	<0.001^e^
Hypertension**	109 (27.95)	107 (27.44)	100 (25.64)	113 (28.97)	1.141	0.767
Dyslipidemia**	79 (20.26)	79 (20.26)	97 (24.87)	131 (33.60)	24.827	<0.001^f^

*Data are presented as mean ± standard deviation; **Data in parentheses are rates (%), and data outside parentheses are counts; ^#^Data outside parentheses are medians, and data inside parentheses are P25 and P75.

^##^Adjusted for multiple testing correction using the Bonferroni method.

^a^Pairwise comparisons among all four groups showed statistically significant differences, P < 0.05;

^b^No significant difference was observed between the Q2 and Q3 groups, while significant differences existed in all other pairwise comparisons, P < 0.05;

^c^No significant difference was found between the third and fourth groups, whereas all other pairwise comparisons showed significant differences, P < 0.05;

^d^No statistically significant differences in MS rates were observed among the groups;

^e^Significant differences existed between Q2 and the other three groups, P < 0.05, with no differences among the remaining groups;

^f^Significant differences existed between Q4 and the other three groups, P < 0.05, with no differences among the remaining groups.

### 3.2 Correlation between TyG and MS-related indicators

Pearson correlation analysis revealed significant associations between the TyG index and multiple metabolic parameters. In the unadjusted analysis, the TyG index showed significant positive correlations with BMI, TG, TC, LDL-C, and FPG (all P < 0.001), while demonstrating a significant negative correlation with HDL-C (P < 0.001). Notably, these association patterns remained statistically significant after further adjustment for potential confounding factors including gender, age, smoking status, and alcohol consumption **Table 2**.

**Table 2:** Correlation between TyG and MS-related indicators.

Indicator	TyG			
	*r*	*P*	r*	P*
DBP	0.04	0.111	0.019	0.457
SBP	0.018	0.481	0.053	0.036
BMI	0.311	<0.001	0.285	<0.001
TG	0.688	<0.001	0.678	<0.001
TC	0.364	<0.001	0.348	<0.001
HDL-C	-0.41	<0.001	-0.393	<0.001
LDL-C	0.183	<0.001	0.155	<0.001
FPG	0.586	<0.001	0.575	<0.001

*Adjusted for gender, age, smoking, and drinking.

### 3.3 Multivariate logistic regression analysis

In the 1,560 subjects, with Q1 as the reference group, multivariate logistic regression analysis was performed after adjusting for age, gender, smoking, and alcohol consumption. The results showed that compared to the Q1 group, the Q4 group had significantly higher risks of MS (OR = 26.994), overweight/obesity (OR = 3.659), and dyslipidemia (OR = 1.929) (P < 0.001) **Table 3**.

**Table 3:** Multivariate logistic regression analysis.

Variable	Crude model (unadjusted)		Adjusted model		Overweight/Obesity		Dyslipidemia	
	OR (95%CI)	P	OR (95%CI)	P	OR (95%CI)	P	OR (95%CI)	P
Q1	1.000		1.000		1.000		1.000	
Q2	3.613 (2.207,5.914)	<0.001	3.711 (2.261,6.092)	<0.001	1.906 (1.420,2.558)	<0.001	1.000 (0.701,1.426)	0.999
Q3	12.988 (8.149,20.701)	<0.001	13.812 (8.606,22.168)	<0.001	2.548 (1.892,3.432)	<0.001	1.300 (0.919,1.841)	0.138
Q4	25.256 (15.817,40.327)	<0.001	26.994 (16.690,43.660)	<0.001	3.659 (2.689,4.978)	<0.001	1.929 (1.371,2.715)	<0.001

In the Adjusted model, Overweight/Obesity, and Dyslipidemia have been adjusted for gender, age, smoking, and drinking.

### 3.4 Predictive value of the TyG index for metabolic syndrome

**Figure 1:**
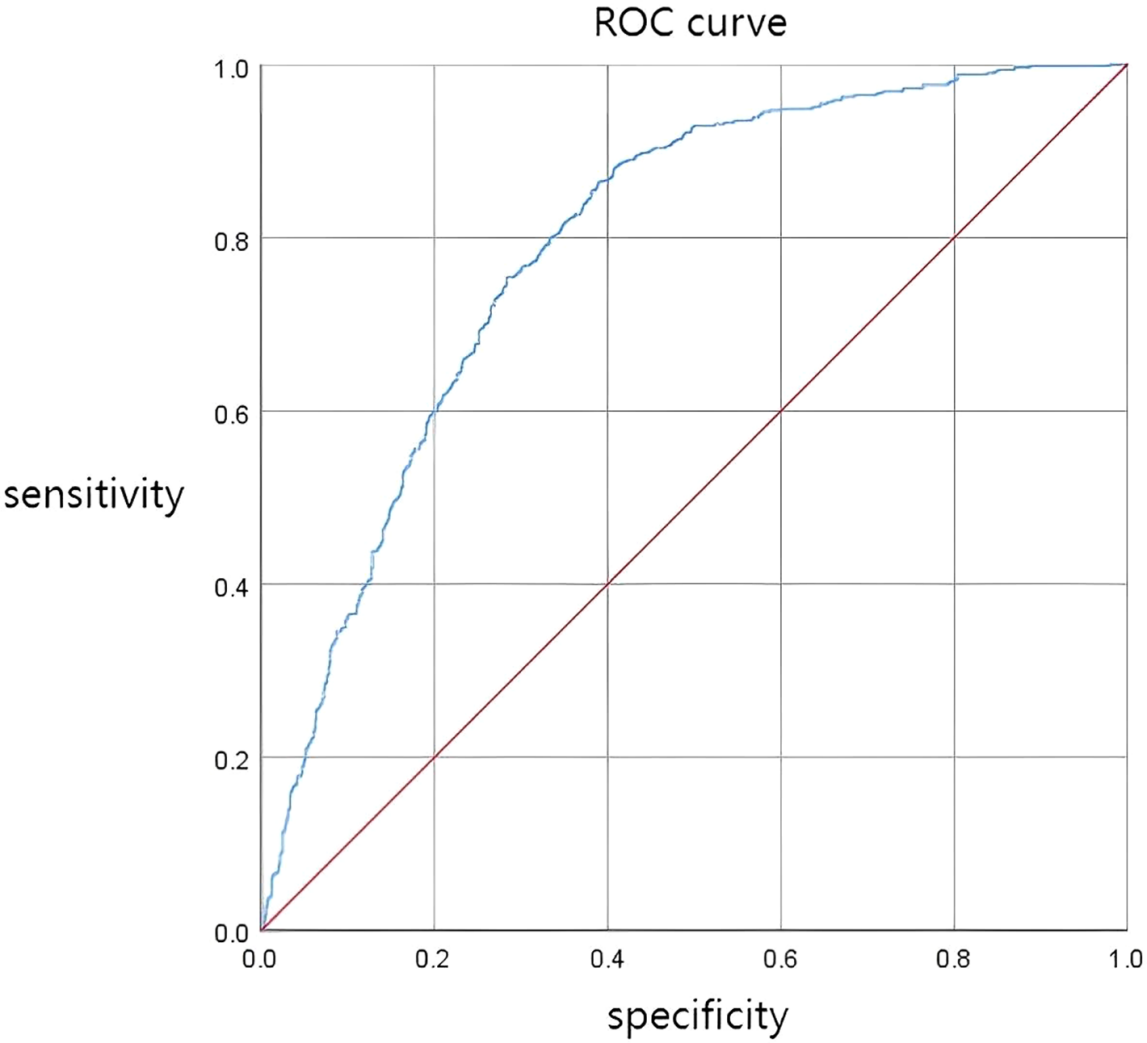
ROC curve for TyG in predicting metabolic syndrome.

ROC curve analysis demonstrated that the TyG index had significant predictive value for metabolic syndrome, with an area under the curve (AUC) of 0.793 (95% CI: 0.770-0.815, P < 0.001),with a sensitivity of 0.864 and a specificity of 0.611 **Figure 1**.

### 3.5 Dose-response relationship between the TyG index and metabolic syndrome

**Figure 2:**
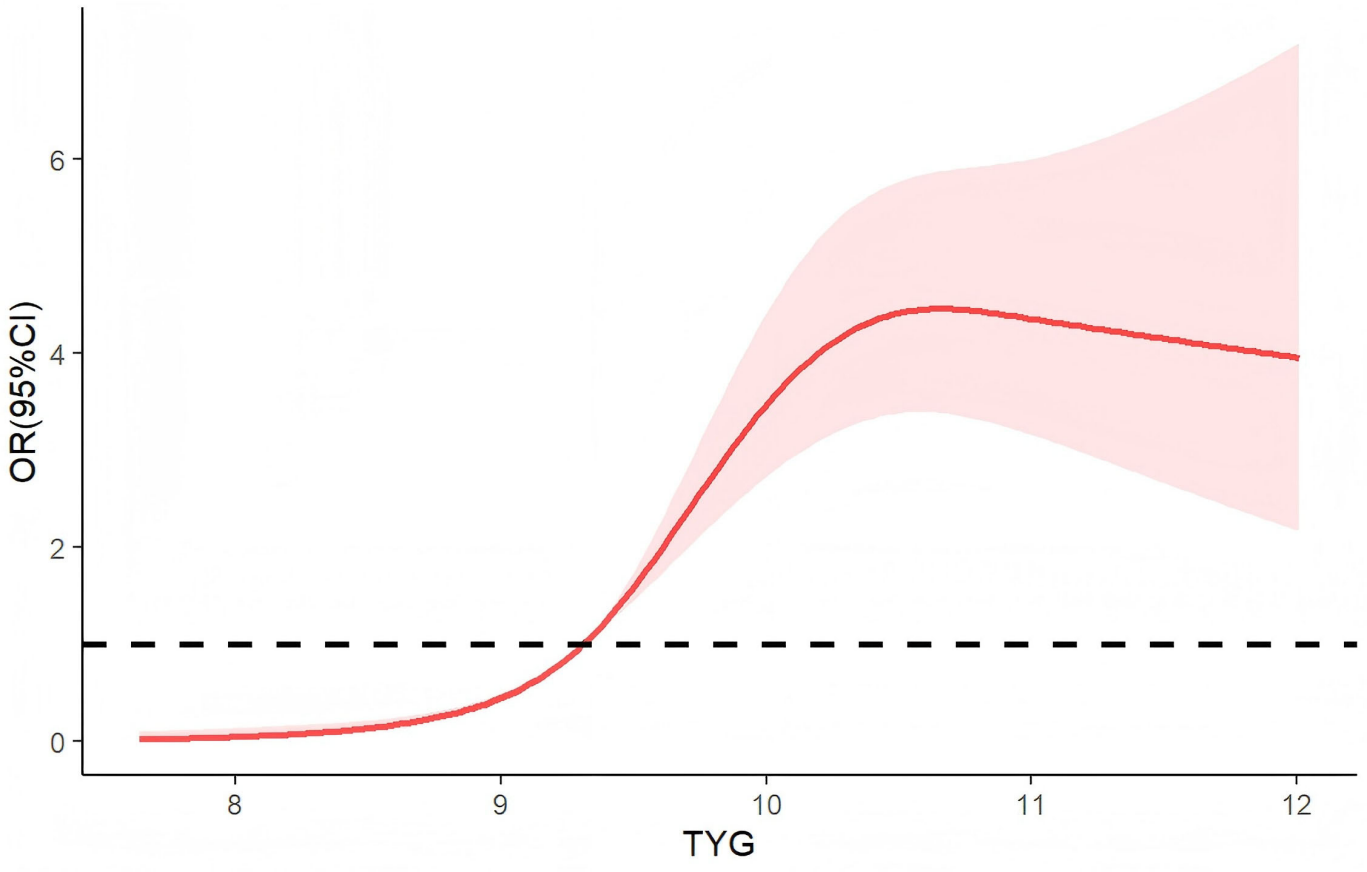
dose-response relationship between the TyG index and metabolic syndrome.

Restricted cubic spline analysis showed that the non-linear model was significantly superior to the linear model (LRT χ² = 70.51, P < 0.001), indicating a non-linear dose–response relationship between the TyG index and metabolic syndrome risk, exhibiting an approximate “inverted U-shaped” curve with an inflection point at 9.31. When the TyG index exceeded 9.31, the risk of MS increased progressively with rising TyG index levels. **Figure 2**.

## 4 Discussion

This retrospective study systematically investigated the association between the TyG index and MS risk in patients with T2DM. The results demonstrated significant correlations between the TyG index and MS as well as its individual components, revealing a distinct nonlinear dose-response relationship. These findings provide important biomarker evidence for MS risk stratification in T2DM patients.

Firstly, we observed that higher TyG quartiles were associated with a gradual increase in the odds of MS, overweight/obesity, and dyslipidemia, even after adjusting for potential confounding factors. Secondly, the TyG index demonstrated excellent predictive ability for MS (AUC = 0.793), indicating its potential as a clinical screening tool. Thirdly, restricted cubic spline analysis revealed a non-linear relationship, with a sharp increase in MS risk when the TyG index exceeded 9.31.

The biological plausibility of our findings is supported by several mechanisms. The TyG index = Ln (fasting triglycerides [mg/dL] × fasting glucose [mg/dL])/2 reflects core lipid (17) and glucose metabolic abnormalities (18) in the pathogenesis of IR. With the progression of IR, impaired insulin signaling in adipocytes leads to increased lipolysis and elevated circulating free fatty acids, which further exacerbate hepatic very-low-density lipoprotein production and hypertriglyceridemia (19–21). Concurrently, progressive pancreatic β-cell dysfunction results in worsening hyperglycemia, while chronic exposure to abnormally high glucose levels adversely affects insulin synthesis/secretion, cell survival, and insulin sensitivity through multiple mechanisms, ultimately leading to sustained deterioration of β-cell function (22, 23). This vicious cycle creates the metabolic milieu captured by the TyG index. Furthermore, the TyG index is closely associated with chronic low-grade inflammation, which serves as a critical bridge connecting IR with various components of MS (24, 25).

Our study has revealed differential associations between the TyG index and components of MS. We found that the TyG index was significantly positively correlated with BMI, TG, TC, and LDL-C, and negatively correlated with HDL-C, which is consistent with previous studies (26–28). The strong correlations with dyslipidemia (TG, TC, LDL-C) and measures of obesity (BMI) may reflect the sensitivity of TyG to lipid metabolic disturbances characteristic of IR. Interestingly, our study found no significant association between the TyG index and blood pressure parameters, compared with Liu et al. (29) (n = 151, mean age 32.11 ± 8.75 years), the present study had a larger sample size (n = 1560), an older population (59.42 ± 11.76 years), and a higher prevalence of antihypertensive medication use, which may explain the absence of a statistically significant association between TyG index and blood pressure. Additionally, this inconsistency may arise from the potential blood pressure-modulating effects of certain antidiabetic medications, such as SGLT2 inhibitors and DPP-4 inhibitors (30–32). There may be a critical metabolic threshold of TyG, beyond which the body’s compensatory mechanisms may be overwhelmed, leading to exponential growth in MS risk. The “inverted-U” shaped curve suggests that when TyG exceeds this threshold, risk stratification and preventive strategies should be intensified.

Our findings have several clinical implications. First, the TyG index represents a cost-effective alternative to complex IR measurements (such as the hyperinsulinemic-euglycemic clamp), particularly valuable in resource-limited settings. Second, the identified TyG threshold can serve as an early warning signal for intensified metabolic monitoring and intervention. Third, our data support incorporating the TyG index into existing risk prediction models to enhance the detection of MS in T2DM patients. Therefore, we recommend that T2DM patients with TyG > 9.31 have their TyG index re-assessed every two years.

Given the chronic nature of metabolic diseases, long-term follow-up data are crucial for assessing the durability of interventions and the long-term prognosis of patients. Existing evidence suggests that clinical outcomes of metabolic disturbances should be evaluated with a follow-up duration of at least ≥5 years to capture endpoint events such as weight rebound, glycemic decompensation, and micronutrient deficiencies (33–35). Studies have shown that the Look AHEAD trial used a weight regain of ≥3% as the criterion for weight rebound, while the long-term follow-up of the Diabetes Control and Complications Trial (DCCT) confirmed (36) that the annual change rate of HbA1c is an independent predictor of microvascular complications.

From a pathophysiological perspective, the TyG index reflects insulin resistance and lipotoxicity - both characteristic of chronic, progressive metabolic dysfunction (37). While our large-scale study with multivariate adjustments has confirmed robust baseline associations, long-term prognostic validation requires prospective study designs. Recent meta-analyses have demonstrated the TyG index’s predictive value for cardiovascular events (38) and diabetic microvascular complications (39), indirectly supporting the clinical relevance of our findings. However, direct verification through longitudinal studies remains necessary.

This study did not evaluate outcomes of bariatric surgery; therefore, differences in surgical techniques do not constitute a confounding factor. Future prospective studies could explore the predictive value of the TyG index in populations with standardized surgical protocols. This study leaves two unresolved limitations. First, its cross-sectional design can only describe the co-occurrence of TyG and metabolic syndrome, but cannot establish which precedes the other. Second, the retrospective data captured medication use crudely (yes/no), making it impossible to disentangle the confounding effects of dose, duration, and adherence. These gaps suggest three priorities for future research: 1.In prospective cohorts with standardized bariatric-surgery or pharmacological protocols, can dynamic changes in TyG prospectively predict the onset or remission of metabolic syndrome; 2.After systematically recording drug regimens and adherence, how will the effect size of the TyG–metabolic syndrome association shift; 3.In a multicenter, larger sample of Chinese patients with T2DM, will the TyG cut-off identified in this study remain robust.

## 5 Conclusion

Our findings position the TyG index as a robust and clinically accessible independent predictor of MS risk in T2DM patients. The strong, graded association and identifiable risk threshold support the potential for integrating TyG into routine metabolic monitoring. Future validation through long-term follow-up studies (≥5 years) is needed to further establish the TyG index’s predictive value for metabolic outcomes and its clinical utility in guiding interventions. Additional research should investigate whether TyG-guided management improves clinical outcomes in this high-risk population.

We are grateful for the financial support from the Sichuan Medical and Health Care Promotion Institute Scientific Research Project(Grant No. KY2023SJ0119); the Jinniu District Health Bureau of Chengdu(JNKY2024-21);the Sichuan Science and Technology Program(2024YFFK0287);and The 2025 Chengdu University Faculty Development Innovation Project (Young Experts Service Corps Special Program). These funding sources played a crucial role in the successful completion of this study.

## Data Availability

The raw data supporting the conclusions of this article will be made available by the authors, without undue reservation.
